# Intraprofessional workplace learning in postgraduate medical education: a scoping review

**DOI:** 10.1186/s12909-021-02910-6

**Published:** 2021-09-07

**Authors:** Lara Teheux, Ester H. A. J. Coolen, Jos M. T. Draaisma, Marieke de Visser, Nynke D. Scherpbier-de Haan, Wietske Kuijer-Siebelink, Janiëlle A. E. M. van der Velden

**Affiliations:** 1 Department of Pediatrics, Radboudumc Amalia Children’s Hospital, Radboud Institute for Health Sciences, Radboud University Medical Center, Nijmegen, The Netherlands; 2 Department of Research on Learning and Education, Radboudumc Health Academy, Radboud University Medical Center, Nijmegen, The Netherlands; 3 Department of Primary and Community Care, Radboud Institute for Health Sciences, Radboud University Medical Center, Nijmegen, The Netherlands; 4 HAN University of Applied Sciences, Nijmegen, The Netherlands

**Keywords:** Intraprofessional learning, Collaborative practice, Workplace learning, Postgraduate medical education

## Abstract

**Background:**

Residents need to be trained across the boundaries of their own specialty to prepare them for collaborative practice. Intraprofessional learning (i.e. between individuals of different disciplines within the same profession) has received little attention in the postgraduate medical education literature, in contrast to the extensive literature on interprofessional learning between individuals of different professions. To address this gap, we performed a scoping review to investigate what and how residents learn from workplace-related intraprofessional activities, and what factors influence learning.

**Methods:**

The PRISMA guidelines were used to conduct a scoping review of empirical studies on intraprofessional workplace learning in postgraduate medical education published between 1 January 2000 to 16 April 2020 in Pubmed, Embase, PsycINFO, ERIC and Web of Science. This study applied ‘best fit’ framework-based synthesis to map the existing evidence, using the presage-process-product (3P) model developed by Tynjälä (2013).

**Results:**

Four thousand three hundred thirty records were screened, and 37 articles were included. This review identified influencing (presage) factors that derived from the sociocultural environment, learner and learning context. Studies described that complexity of care can both facilitate and hinder learning. Furthermore, intraprofessional learning is threatened by professional stereotyping and negative perceptions, and awareness of learning opportunities and explicit reflection are critical in intraprofessional workplace learning. Studies described a range of informal and formal intraprofessional activities (process) under the headings of collaboration in clinical practice, rotations or placements, formal educational sessions and simulated workplace training. In general, learners responded well and their attitudes and perceptions improved, learners reported increased knowledge and skills and positive behavioural changes (product). Learning outcomes were reported in the domains of patient-centred care, collaborative attitudes and respect, mutual knowledge and understanding, collaborative decision making, communication, leadership, teamwork and reflexivity.

**Conclusions:**

This review gives insight into the high learning potential of intraprofessional activities. Many of the included studies relied on self-reported perceptions of change, therefore, future research should focus on generating more robust evidence including objectively examined outcome measures. This review offers a comprehensive overview of the factors that influence intraprofessional workplace learning in postgraduate medical education. Finally, we provide recommendations for enhancing intraprofessional learning in clinical practice.

**Supplementary Information:**

The online version contains supplementary material available at 10.1186/s12909-021-02910-6.

## Background

Modern patient care is highly complex; the exponential growth of medical knowledge and technological advances result in a high degree of specialization, requiring health professionals from various disciplines to collaborate effectively in order to achieve high-quality patient care [1–4]. Effective interdisciplinary teams improve patient outcomes and reduce costs by diminishing service duplication, unnecessary interventions, and complications [2, 5]. Consequently, the need to train residents to work and learn effectively in interdisciplinary teams has received considerable attention in educational policies and accreditation standards [5–8].

A substantial amount of literature has been published on interprofessional learning, which is defined as the learning that occurs when two or more professions engage [8]. Literature reviews describe that interprofessional education leads to a positive change in attitudes and increased knowledge and skills required for collaborative practice [9–14]. Additionally, the findings from several heterogenous studies suggest that interprofessional education may have a positive effect on patient care outcomes by overcoming communication barriers within medical hierarchy, decreasing tensions, and enhancing understanding of each other’s roles and expertise [12–14]. Furthermore, previous research has established an understanding of the multitude of factors that enable or hinder interprofessional learning [2, 10, 12, 15–18].

Surprisingly, to date little attention has been paid to intraprofessional learning [19] (i.e. the learning that occurs when individuals of two or more disciplines within the same profession engage [8]). While the principles of intra and interprofessional learning are similar at a core level, there are specific differences related to the practices within the medical profession and the relationships between doctors of different specialties and between primary and secondary care doctors, that merit further investigation of intraprofessional learning [8]. In the light of increasing specialization and patient care complexity, intraprofessional learning has become imperative as no one doctor can meet all complex patient care needs, and establishing effective communication between medical specialties — with their own vocabulary, approaches, and understandings — has become increasingly challenging [2, 4, 20].

Intraprofessional learning is of particular importance in postgraduate training as discipline-specific ‘cognitive maps’ (i.e. the whole cognitive and perceptual approach of a discipline, which is a major component of a discipline’s culture) are developed and reinforced through the socialization process of educational experiences, and gaining adequate understanding of each other’s cognitive maps is an important challenge in intraprofessional collaboration [2, 4, 20, 21]. Postgraduate training programs often include multiple intraprofessional rotations, during which residents are exposed to the distinct cultures and practices of various specialties, creating a period of high intraprofessional learning potential. On the one hand, the intraprofessional encounters in residency training can create ‘productive’ tensions in conversation and collaboration between health professionals that can promote learning through experiencing and dealing with differences in cognitive maps, power differentials, pushback and uncertainties [10, 20, 22–24]. On the other hand, tensions between health professionals may also be ‘unproductive’ and impair learning as residents may lose the desire to understand the perspective of the other if the tensions are perceived as too unpleasant [2, 22–24]. These productive and unproductive tensions first emerge during postgraduate training, as this is the first time that doctors work and learn in separate groups [2]. Therefore, unravelling the process of intraprofessional learning in postgraduate training is of utmost importance to our understanding on how to prepare a “collaborative practice-ready” health workforce [5].

For these reasons, a generalized overview of the existing literature on intraprofessional learning in postgraduate medical training is overdue. Therefore, we conducted a scoping review. To our knowledge, this is the first review to explore intraprofessional workplace learning in postgraduate medical training. We chose a scoping review approach, as this methodology is particularly helpful in studying literature in research areas with emerging, heterogenous evidence [25–27]. As postgraduate medical training is situated at the workplace [28], we decided to focus our review on the intraprofessional learning related to the workplace (i.e. the settings where residents work including hospital and community settings). With this scoping review, we aimed to describe and evaluate existing literature in order to advise educational policy makers, program directors and intraprofessional teams on how to enhance intraprofessional learning in the workplace, as well as to identify areas for future research. The following two research questions were formulated: (1) What and how do residents learn from workplace-related intraprofessional activities? (2) What factors influence intraprofessional workplace learning in postgraduate medical training?

## Methods

### Study design

This study adopted a scoping review approach. The scoping review (or scoping study) is a strategy designed to map literature in a research area, identifying key concepts, sources of evidence, and research gaps [25–27]. We employed the commonly-used methodology proposed by Arksey and O’Malley [25] and advanced by Levac, Colquhoun and O’Brien [26]. To further ascertain the methodological quality of this review, we employed the PRISMA Extension for Scoping Reviews [27]. The scoping review protocol was registered in the Open Science Framework (https://osf.io/p9xf6).

### Selection of studies

The eligibility criteria are summarized in Table 1. We considered workplace learning to encompass incidental and informal learning, intentional non-formal learning, and formal on-the-job and off-the-job training, in order to assimilate the full extent of resident learning related to the workplace environment [30]. We included papers published in peer-reviewed journals with empirical data. We decided to exclude grey literature, as including this would result in an unfeasible number of documents and we felt it would not compromise the answer to the research questions given the breadth of articles represented in peer-reviewed journals. Commentaries, reviews, books, and papers focused on description of curricula were excluded, due to the lack of a research component. Literature published before the year 2000 was excluded; the results from these studies were not considered recent enough as the beginning of the twentieth century marked a reform in health professions education and an increased interest in inter and intraprofessional education [3, 31].

**Table 1:** Eligibility criteria used in this review

Inclusion criteria	Exclusion criteria
Focus on intraprofessional learning, i.e. the learning that occurs when two or more disciplines of the same profession engage [8].	Does not meet inclusion criteria of focus on intraprofessional learning, primary and/or secondary care postgraduate medical trainees and workplace learning.
Involves primary and/or secondary care postgraduate medical trainees ﻿[29].	Grey literature.
Workplace learning: incidental and informal, intentional non-formal, and/or formal [30].	Reviews, commentaries, book, papers only describing curricula (no empirical data).
Contains empirical evidence from qualitative, quantitative or mixed methods studies.	Publication before 2000.
Published in a peer-reviewed journal.	Written in another language than English.
	Unable to retrieve abstract or full-text paper.

We searched 5 electronic databases (PubMed, Embase, PsycINFO, ERIC and Web of Science) using the following Boolean search strategy identified through input from the research team and consultation of the university-affiliated librarian: postgraduate medical education AND intraprofessional AND learning and education and their synonyms. Both subject headings (such as MeSH) and free text terms were applied. Search results were limited by English language and publication date from 2000. A sample search strategy (for PubMed) is provided in Additional file 1. The initial search was performed on April 16, 2020. Search results were collected and deduplicated in Endnote and then exported into Rayyan software [32] for ease of management.

The first author (LT) and a second reviewer (JD, MV or JV) independently screened all article titles and abstracts to determine eligibility for full text review. Discrepancies between reviewers were resolved by discussion and consensus or involvement of a third reviewer. Full texts of all remaining studies were retrieved and eligibility was assessed independently by LT and a second reviewer (EC, JD, WK or JV*)* based on the same criteria and methods applied in title and abstract screening. Reference lists of reviews and included studies were scanned to supplement the search, using the same methods and criteria.

### Data analysis

No methodological quality assessment was performed, as we aimed to map all existing evidence on intraprofessional learning in the workplace in postgraduate medical education and not to present a judgement regarding the ‘weight’ of evidence [25].

For the numerical descriptive summary, an initial data extraction chart was drafted collectively by the research team and tested independently by two reviewers (LT and EC or WK) in a random sample of 10 articles.

For the qualitative analysis and synthesis of the evidence, we applied ‘best fit’ framework-based synthesis, which allows themes that were identified a priori to be specified as coding categories for deductive analysis, and to be combined with de novo concepts following from inductive analysis [33–35]. We chose this method as it allows previously established theoretical frameworks to be explicitly and systematically considered in the analyses rather than generating theories de novo, while also maintaining enough flexibility to *inductively* detect new themes that emerge from the data. This method is considered especially useful when relevant theories exist but have not been refined in the specific context of the research question [34, 35].

The research team developed the a priori framework based on reflection upon the experiences of the pilot and pre-existing frameworks on inter- and intraprofessional learning and workplace learning in the literature [3, 36–38]. The theoretical framework is described below. A list of themes was derived from the theoretical framework and constituted the a priori framework of themes used to code the data from the included studied. The first author (LT) chartered the data and coded all included articles line by line. When relevant data did not fit in any of the a priori themes, additional themes were inductively added after discussion by at least two authors (LT and EC, JD, WK or JV). Regular meetings of the research team during the data analysis facilitated critical discussion of the data. After reviewing all included studies, the research team discussed the data extraction chart and themes, both from the a priori framework and the inductive thematic analysis, to reach consensus on the final themes and framework to be reported.

#### Theoretical framework

As learning in the workplace is central in postgraduate medical training [28], we employed Tynjälä’s 3-P (presage-process-product) model of workplace learning [36] as an analytical framework to synthesize the data from all the included studies, in order to untangle the complex phenomenon of intraprofessional learning in relation to the sociocultural environment, learner and context factors, learning processes and learning outcomes. Tynjälä regards *presage factors* as the learning context (i.e. relating to work organisations and their features) and the characteristics of individuals who participate in the learning, also recognizing the importance of the learner’s interpretation of the presage factors. The *process* component describes the different work activities through which learning processes take place and the *product* component represents the learning outcomes. These three components are attached to the *sociocultural environment,* which reflects the sociocultural context in a wider sense (beyond the specific local context) and encompasses all artefacts of human culture, including the technical-organisational environment. The sociocultural environment is placed as a surrounding frame as it plays a determining role in the *presage, process* and *product* of workplace learning.

In order to gain a more detailed understanding of the diversity of learning outcomes (product) specific to collaborative practice in medical care, we decided to enrich the product section of our analytical framework with the competency frameworks from Janssen et al. [37] and Rogers et al. [38]. These authors defined a number of competencies relevant for collaborative care: ‘patient-centred care’, ‘roles and responsibilities’, ‘role understanding’, ‘mutual knowledge and understanding’, ‘collaborative attitude and respect’, ‘interprofessional values’, ‘communication’, ‘teamwork’, ‘leadership’, and ‘reflexivity’. Lastly, we adopted the four level learning outcome typology originally designed by Kirkpatrick [39] as operationalized in the interprofessional learning continuum (IPLC) model [3]. In this model, learning outcomes are classified in four non-hierarchical levels: learner’s reactions (level 1); changes in attitudes or perceptions (level 2a); acquisition of knowledge or skills (level 2b); behavioural change (level 3); and performance in practice (level 4). We felt that this typology would be helpful to facilitate the narrative about the outcome measures used in these studies and to illuminate research gaps, in order to inform policy development and areas for future research.

### Research team

The research team was composed of members with diverse backgrounds and experience in postgraduate medical education and educational research: a medical doctor and PhD student in postgraduate education (LT), a senior educational researcher and lecturer in the field of interprofessional education (WK), a senior educational researcher and educationalist (MV), a general practitioner and director of primary care specialty training (NS), and three paediatricians with experience as program director of paediatric specialty training (JD, EC, JV).

## Results

**Fig. 1:**
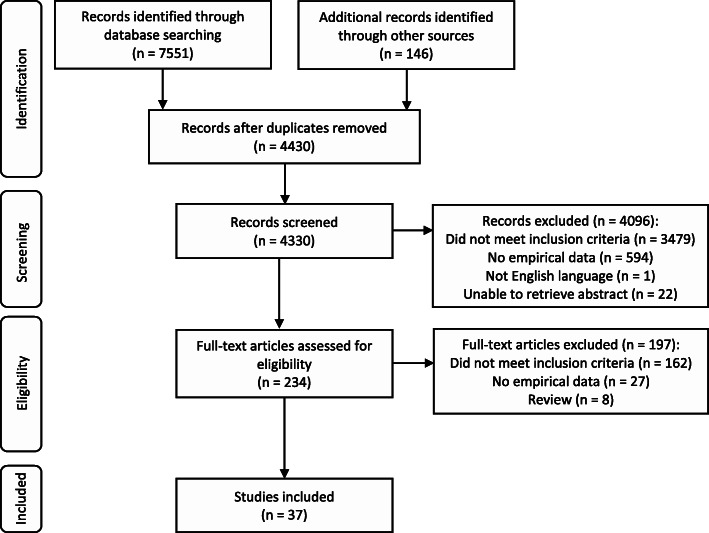
PRISMA flow diagram

Figure 1 displays the PRISMA flow diagram. The electronic database search retrieved 7551 citations, and the reference lists of reviews and included articles provided 146 extra citations. Four thousand three hundred thirty records were screened for eligibility. Finally, we included 37 articles describing 35 unique studies in the review.

The included studies were heterogenous in design. Fourteen studies employed qualitative methods (38%) [40–53], 13 studies used quantitative methods (35%) [54–66], and 10 studies had a mixed methods approach (27%) [67–76] (see Table 2). Of the quantitative and mixed methods studies, 11 studies [54–56, 58, 59, 61, 62, 71–74] employed a pre/post-test design. All qualitative studies [40–53] and 6 of the mixed methods studies [67, 68, 71–73, 75] used interviews as one of the methods for data collection, in some studies triangulated with other methods such as observations or document analysis [42, 44, 52, 71].

**Table 2:** Summary of study methodology, learning typology, learning activities and learning outcomes

	Number (percent) of articles	References
**Study methodology**		
Qualitative	14 (38%)	[40–53]
Quantitative	13 (35%)	[54–66]
Mixed methods	10 (27%)	[67–76]
**Learning typology**		
Informal/nonformal learning	15 (41%)	[40, 42, 44–49, 51–53, 58, 60, 68, 70]
Formal learning	8 (22%)	[56, 59, 62, 64, 71, 72, 74, 75]
Combination of informal/nonformal and formal learning	10 (27%)	[41, 43, 50, 54, 55, 61, 65, 69, 73, 76]
**Reported learning activities**		
Collaboration in clinical practice	14 (38%)	[40, 42, 44–46, 49, 51–54, 57, 60, 69, 70]
Consultations	5 (14%)	[40, 42, 51–53]
Radiology rounds	2 (5%)	[60, 70]
Combined outpatient clinic	1 (3%)	[54]
Co-management inpatient ward	1 (3%)	[69]
Rotations or placements	8 (22%)	[48, 50, 55, 58, 61, 65, 68, 76]
Formal educational sessions or programs	11 (30%)	[41, 43, 56, 59, 62, 64, 71–75]
Simulated workplace training	4 (11%)	[56, 64, 71, 75]
**Reported learning outcomes**		
Level 1: learner reactions	24 (65%)	[40, 43, 46, 48, 50, 51, 53–58, 60, 62, 64, 65, 68–75]
Level 2a: changes in attitudes or perceptions	21 (57%)	[43, 46, 48, 50, 51, 55, 56, 59–62, 64, 65, 68, 70–76]
Level 2b: acquisition of knowledge or skills	24 (65%)	[40, 43, 46, 48, 50–52, 54–56, 58–62, 65, 68–70, 72–76]
Level 3: behavioural changes	8 (22%)	[43, 46, 51–53, 71–73]
Level 4: performance in practice	0 (0%)	

A broad range of medical specialties were involved in the intraprofessional activities described in the included studies. The specialties most often involved were internal medicine (*n* = 16) [42, 44, 46, 47, 51–54, 60, 61, 65–67, 70–72], surgical specialties (*n* = 14) [46, 47, 49, 52, 53, 56, 57, 59, 63, 64, 69, 71, 72, 75], paediatrics (*n* = 11) [41, 43, 45, 47, 48, 50, 54, 61, 68, 69, 76], family medicine (*n* = 11) [40, 41, 43, 44, 46, 51, 55, 58, 71–73], emergency medicine (*n* = 10) [42, 48, 50, 52, 53, 56, 59, 68, 71, 72] and geriatrics (*n* = 9) [52, 53, 55, 61, 65–67, 72, 74]. The complete dataset is available in the DANS EASY repository, 10.17026/dans-zb5-2hfg.

**Fig. 2:**
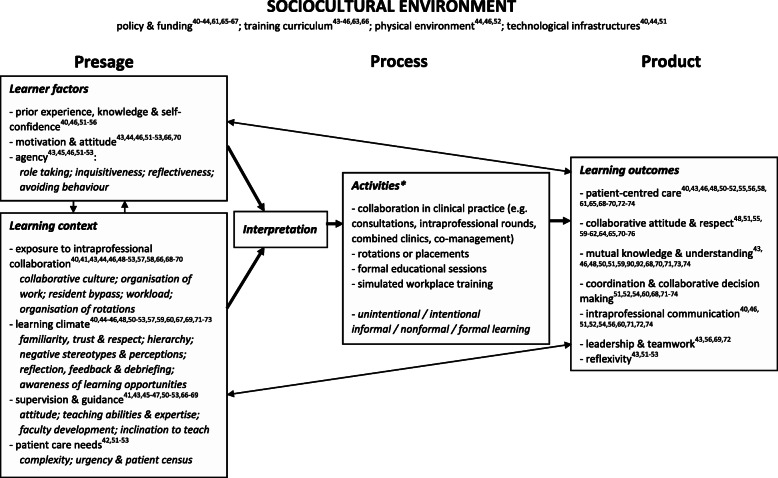
The 3-P model of intraprofessional workplace learning (modified from Tynjälä 2013). * An overview of the references for process factors is provided in Table 2

Figure 2 presents a summary of our findings in a modified 3-P model [36]. Below we describe the themes found in four main sections: sociocultural environment, presage, process, and product.

### Sociocultural environment

Many of the intraprofessional activities described in the included studies were initiated due to influences from the sociocultural environment, such as changes in national or regional policy, training curriculum requirements or availability of a grant (e.g. [61, 65–67]). While many of the stimuli from the sociocultural environment were described as positive influences, Webster et al. [42] provide an insightful account of a policy change that resulted in enhanced focus on efficiency at the emergency department, which came at the expense of learning opportunities for residents and caused tensions between specialties [40, 41, 43, 44, 66, 67]. Furthermore, several studies reported a lack of reimbursement for intraprofessional care activities and a lack of preparedness of the care system for integrated care to be a barrier to intraprofessional collaboration and learning in practice [40, 41, 43, 44, 66, 67].

The embedding of intraprofessional learning in training curricula was found to be a key influencing factor [43–46, 63, 66]. Nevertheless, studies observed that collaboration competencies are not formalized in the training curriculum and learned informally on the job [44–46]. Integration in training curricula is complicated by conflicting time demands with other curricular requirements, significant variability across residency programs and malalignment of competency frameworks, and lack of priority on the academic agenda [44, 46, 63, 66]. Griffin et al. [43] suggest that a lack of interprofessional education in the undergraduate curriculum may make it difficult to adopt the competencies required for integrated care later in the career.

The physical environment is an important determinant of the possibilities and limits of intraprofessional workplace learning [44, 46, 52]. Specifically to the learning at the interface between primary and secondary care, studies reported that being in physically distant locations resulted in less interaction between primary care trainees and medical specialists [44, 46]. Furthermore, hospital size affected learner roles; in smaller hospitals doctors learn more by being responsible and treating patients independently, while in larger hospitals they benefit from highly specialized knowledge from experts but acted less independently in patient care [52].

Three studies that investigated collaboration between primary and secondary care doctors reported that technological infrastructures (e.g. electronic patient records or referral systems) can facilitate or constrain intraprofessional collaboration and learning [40, 44, 51]. These challenges were not reported in within-hospital settings.

### Presage

#### Learner factors

Residents’ prior experience, knowledge, and self-confidence shape intraprofessional communication and learning [40, 46, 51–56]. In general, studies described that self-reported learning was greater in trainees with less experience or prior knowledge and that less experienced learners mainly learn through observing, questioning and deliberate teaching by experts, while experienced learners demonstrate more self-directed learning and take more responsibility in patient care [52–56]. Learner insecurity or uncertainty lead to initiation of doctor-to-doctor consultations and more extensive intraprofessional discussion, whereas high self-perceived knowledge resulted in more brief communication and a smaller likelihood of consulting another specialty [46, 51, 53]. Several studies suggested that the learning from intraprofessional consultations may result in a diminished need for consultations or shifting referral questions [40, 51, 53].

Other frequently reported learner factors were motivation and attitude [43, 44, 46, 51–53, 66, 70]. High learner motivation encourages residents to seek intraprofessional interactions, thereby increasing the learning effect [51, 52]. Learning relevant skills for patient care and contributing to high quality patient care are important motivators for intraprofessional collaboration [43, 44, 46]. Four studies reported that residents were more motivated to learn intraprofessionally with specialties more closely related to their intended specialization [52, 53, 66, 70].

Factors related to learner agency (i.e. the intentionality and actions of the trainee that mediate learning [77]) were reported in a number of studies investigating intraprofessional activities [43, 45, 46, 51–53]. The level of self-directedness was determined by the role taken by the learner, and learners with an inquisitive nature were more likely to initiate intraprofessional consultations [43, 51, 52]. Explicit learner reflection was found to be highly valuable for the learning process [46, 52, 53]. Important triggers for reflection were potential errors and situations in which the views of the learner deviated significantly from the ideas of the expert [53]. One study reported avoiding behaviour after conflicts in collaboration [45], which likely had a negative impact on learning.

#### Learning context

##### Exposure to intraprofessional collaboration

A key factor in the learning process was the level of exposure to intraprofessional collaboration. The collaborative culture was one of the determinants of intraprofessional exposure [40, 41, 44, 51, 52, 57, 68, 70]. Face-to-face contact was preferred as this contributes to an intraprofessional mindset [40], whereas limited interaction was found to restrict learning opportunities [40, 41, 44, 52, 57, 70]. Multiple studies addressed that there was a culture of working separately between specialties, e.g. not including the other specialty in consultations [40, 41, 44, 57]. It was suggested that this may be due to isolation of work settings or a general lack of awareness of the need for collaboration [44, 57].

We found numerous descriptions of how the organisation of work influenced residents’ exposure to intraprofessional collaboration [40, 44, 46, 48, 50–53, 58, 66, 69, 70]. A decisive factor was resident task assignment; whether or not trainees have the opportunity to learn in intraprofessional teams (e.g. in the role of requesting or responding doctor in intraprofessional consultations [40, 44, 46, 51–53]) depends on organisational structures, such as who carries the pager for consultations and who is invited to multidisciplinary meetings [46, 52]. Furthermore, task assignment may enable exposure to certain patient groups and teaching opportunities with subspecialist faculty [58, 69]. Additionally, day-time or night-time shifts also affect exposure to intraprofessional consultations, as in some settings consultations only happen during day-time hours [46, 52].

Exposure to intraprofessional collaboration is threatened by ‘resident bypass’ in intraprofessional care by supervisors [49].﻿ Reported reasons for resident bypass included lack of resident specialized knowledge, frequent resident transitions, concerns about the quality of communication or inadequate decision making by residents, and the urgent nature of consultations [49].

Multiple studies reported that high workload or a lack of time was a major barrier to intraprofessional learning, as it diminishes opportunities for direct contact and relationship building, interferes with residents’ exposure to intraprofessional activities such as courses, and limits accessibility and availability of supervisors or consultants [40, 43, 44, 46, 48, 53, 66]. Furthermore, the organisation of intraprofessional rotations (e.g. selection of suitable patient care activities [58], side-by-side integration of subspecialties [50]) affected exposure and, thereby, intraprofessional learning.

##### Learning climate

The interaction between specialties plays an important role in establishing the open communication, familiarity, trust and respect that contribute to a fruitful learning climate [46, 50–52, 69, 71–73]. Intraprofessional contact was found to be easier between residents of equal training level as this leads to little experienced hierarchy [51, 71]. Studies observed that intraprofessional rotations, consultations, co-management models, and formal training courses could build trust and a sense of belonging [50, 69, 72, 73], although one study observed that this trust returned to the initial level after 3 months, suggesting that maintaining contacts is necessary for a long-lasting effect [73].

Intraprofessional collaboration and learning was hindered by stereotypes and negative perceptions towards the abilities of the other specialty [40, 44, 51, 57]. These obstacles to collaboration and learning were more pronounced in studies investigating learning between primary and secondary care doctors [40, 44, 51]. Beaulieu et al. [44] reported illustrative examples of stereotyped negative behaviour and poor role modelling by supervisors (e.g. advising action without consulting the referring specialty), suggesting that this may be caused by the differentiation of identities between general practitioners and specialists in postgraduate training as supervisors seemed less reflective about their role in intraprofessional collaboration than residents.

Multiple studies addressed that time for reflection and debriefing were highly important for learning [40, 45, 50–53, 67]. Specific to consultations, studies noted that the absence of a feedback mechanism from referrer to consultant (e.g. on the appropriateness or helpfulness of the advice) was a missed learning opportunity [40, 51]. Another reported factor related to the learning climate, was the awareness of intraprofessional learning opportunities [45, 46, 48, 53, 59, 60, 67, 71]. Awareness and interest in intraprofessional learning at the workplace was stimulated by formal intraprofessional activities such as courses or placements and could be fostered by labelling learning opportunities and debriefing conflicts [45, 46, 48, 59, 60, 71].

##### Supervision and guidance

Supervisors were described to influence intraprofessional learning in a number of ways. First, supervisors’ attitude could facilitate or hinder intraprofessional learning through encouraging or discouraging residents to engage in intraprofessional conversations [46, 51, 66].

Second, a number of studies addressed the importance of supervisor’s teaching abilities to facilitating resident learning and creating a safe learning environment [45, 47, 52, 53, 67, 68]. A supervisor’s ability to provide intraprofessional guidance depended on their level of expertise and experience, and familiarity with the clinical context of the learner [47, 68]. Three studies addressed supervisors’ lack of training or knowledge on how to teach collaboration competencies [41, 43, 45], stressing the need for faculty development in this area.

Third, side-by-side supervision by experts from different specialties was found to enrich resident learning by providing different perspectives and approaches [68, 69], whereas supervision by a limited number of preceptors specialized in specific areas may limit development in other areas [50].

Last, studies described that supervisors’ inclination to teach residents is dependent on other factors such as work load and time of day [46, 52].

##### Patient care needs

A number of studies investigating intraprofessional consultations described how patient care needs influenced intraprofessional learning [42, 51–53]. Respondents in the included studies indicated that they learned most from complex cases as these required interaction with experts, while less complex care would stimulate more autonomous working and learning [51–53]. Furthermore, studies described that urgency and high patient census negatively impacted learning, as it limited time for self-directed examination, clinical reasoning, and teaching by experts [42, 52].

### Process

Studies reported on a range of intraprofessional activities, summarized in Table 2. The majority of studies investigated intraprofessional collaboration in clinical practice in which residents acted in the role of the own specialty [40, 42, 44–46, 49, 51–54, 57, 60, 69, 70]. Several studies addressed that collaboration competencies are not formally taught, but learned informally in the context of patient care [44–46]. Described learning processes include being responsible for patients in intraprofessional care, questioning and discussion with experts, role modelling, coaching, and feedback [45, 46, 53].

A number of studies reported on intraprofessional rotations or placements in which residents acted in the role of another specialty or shadow someone from another specialty [48, 50, 55, 58, 61, 65, 68, 76]. The rotations often included a combination of formal educational sessions and informal/nonformal learning. The duration of the rotations varied between 3 weeks to 3 months.

The duration of reported workplace-related formal education activities varied between less than 1 day to numerous sessions in a year-long program [41, 43, 56, 59, 62, 64, 71–75]. See dataset for more details, 10.17026/dans-zb5-2hfg.

### Product

#### Outcome measures

The reported learning outcomes are categorised according to Kirkpatrick’s levels [3, 39] in Table 2. Level 1 learning outcomes typically addressed participants feedback related to the level of: satisfaction with the activity, appreciation of intraprofessional learning, or usefulness or enjoyment of the activity. In general, this data was obtained through Likert-scale or open-ended survey questions or interviews.

Level 2 learning outcomes were often evaluated using surveys or interviews reporting self-assessment of changes. Knowledge and skills were often assessed in terms of confidence, self-efficacy or level of comfort, and in the majority of studies the applied measures had not been previously validated. An example of a study that gathered more robust data at these levels was reported by Faulk et al. [55], who employed a pre/post-test design to investigate changes in attitudes and knowledge with pre-validated instruments and performed a baseline comparison of knowledge in a control group. Another example is the study by Bullard et al. [71], who gathered attitudinal data in an intervention and control group using a pre/post-test design, triangulated with field observations and interviews, although this study utilized an unvalidated survey.

All of the studies that reported on behavioural change had a qualitative or mixed methods approach. The mixed methods studies describing level 3 outcomes often employed a longer follow-up period than the studies that only reported level 1–2 outcomes [72, 73]. All studies that addressed behavioural change mainly drew on self-reported perceptions of change. Four studies also investigated resident behavioural change from the perspective of supervisors [43, 51–53]. None of the studies reported learning outcomes on level 4.

The reported learning outcomes will be thematically discussed in the following paragraphs.

#### Patient-centred care

Fourteen studies (38%) reported positive learning outcomes in the domain of patient-centred care [40, 43, 46, 48, 50–52, 56, 61, 68–70, 73, 74], four studies (11%) reported mixed outcomes (combination of significant positive and non-significant findings) [55, 58, 65, 72], and no studies reported negative outcomes. The majority of these studies described outcomes related to increased confidence in one’s ability to provide care for certain patient groups that were shared between specialties, or enhanced knowledge or skills relevant for the care of the shared patient groups [40, 46, 48, 51, 52, 55, 56, 58, 61, 65, 68–70, 72–74] (level 2b). One study reported a better understanding of the unique needs of older patients and self-assessed improved patient management in clinical practice in the 12-month follow-up after a geriatrics course [72] (level 3). Other reported outcomes included improved attitude [55] (level 2a) and increased confidence in teaching peers [72] (level 2b) regarding specific patient groups.

Often, the learning described in these studies was unidirectional: residents from one specialty would learn knowledge or specific skills from another ‘expert’ specialty, while no learning by the ‘experts’ would be reported. A small number of studies did report reciprocal learning in which both specialties would gain a better understanding of the patients [46, 51, 70, 73].

Two studies reported that an intraprofessional program stimulated patient-centred practice [43, 50] (level 2a/3). It is uncertain if these learning outcomes should be attributed to the intraprofessional set-up or to the content of the programs, as the content of these programs was more explicitly focused on patient-centred or integrated care than programs described in the other studies.

#### Collaborative attitude and respect

Fourteen studies (38%) described a positive change in collaborative attitudes [48, 51, 59–62, 64, 70–76], and two studies (5%) described mixed outcomes (combination of significant positive and non-significant findings) [55, 65]. Overall, the studies reported improved attitudes towards teamwork, enhanced appreciation and understanding of the importance of collaboration, and increased enjoyment in intraprofessional collaboration [48, 51, 55, 59, 62, 71, 73, 75, 76]. Additionally, studies found that working relationships improved through development of trust, mutual respect, and enhanced awareness of common goals [51, 59, 64, 70–73]. It should be noted that all of the studies used self-report techniques, none of the studies reported on attitudes as experienced by the other specialty (level 2a).

#### Mutual knowledge and understanding

We found 14 studies (38%) that described outcomes related to mutual knowledge and understanding [43, 46, 48, 50, 51, 59, 60, 62, 68, 70, 71, 73, 74]; all reported positive outcomes. These studies provided evidence that collaboration in clinical practice (e.g. through consultations and intraprofessional rounds) and intraprofessional rotations helped residents to understand the practice environment, expertise and needs of the other specialty [43, 46, 48, 50, 51, 59, 60, 62, 68, 70, 71] (level 2a/b). Other reported outcomes included self-reported increased knowledge on how and when to consult the other specialty, insight into how advice affects the other specialty and better understanding of consultation requests or reports of the other specialty (level 2b) [46, 48, 51, 59, 60, 62, 70, 74].

#### Coordination and collaborative decision making

Nine studies (24%) reported positive outcomes related to coordination and collaborative decision making [51, 52, 54, 60, 68, 71–74] (level 2a/b and 3). Reported level 2 outcomes included learning procedural knowledge regarding coordination of care, feeling more prepared to communicate patient care needs, and self-reported improved consultation skills [52, 54, 60, 68, 73, 74]. Four studies reported level 3 outcomes [51, 71–73]. Respondents in an interview study indicated that their positive experiences in a consultation program stimulated them to seek collaboration, and supervisors noted that residents learned to take responsibility and act independently [51]. In the 18-month follow-up after a joint program for general practitioner and occupational health trainees, participants reported enhanced awareness of ‘the process of cooperation’, more initiative to contact each other, better coordinated policy, and a clearer division of tasks in clinical practice [73]. In the mixed methods studies by Bullard et al. [71] and Levine et al. [72], respondents noted that a multidisciplinary course eased interactions and improved residents’ ‘ability to acknowledge other clinical perspectives’ in clinical practice.

#### Intraprofessional communication

Learning outcomes related to intraprofessional communication were reported in nine studies (24%) [40, 46, 51, 52, 54, 56, 60, 71, 72, 74]; these included feeling more prepared or confident to communicate with other specialties, learning how to articulate consultation requests and reports, and learning how to tailor communication to other specialties (level 2a/b). Improvement of intraprofessional communication in clinical practice was reported in three studies [46, 71, 72] (level 3); these studies all employed only self-reported perceptions of change.

#### Leadership and teamwork

Four studies (11%) reported on learning outcomes related to leadership and teamwork skills [43, 56, 69, 72]. Reported outcomes included feeling more prepared for interprofessional teamwork [69] (level 1) and enhanced confidence in leadership and teamwork skills (e.g. leading a resuscitation team, group facilitation, conflict resolution) [56, 72] (level 2b) [56, 69, 72]. In their interview study, Griffin et al. [43] reported that mentors and trainees felt that an integrated care program enhanced leadership skills in patient care (level 3).

#### Reflexivity

Four studies (11%) addressed learning outcomes related to reflexivity; intraprofessional interaction was found to stimulate reflection, taking responsibility and being critical about your own questions [43, 51–53], (level 3).

## Discussion

The aim of this scoping review was to explore what and how residents learn from workplace-based intraprofessional activities, and what factors influence intraprofessional workplace learning. We included 37 articles, which reported on a range of intraprofessional activities and represented a broad spectrum of medical specialties in primary and secondary care.

### Learning outcomes

This review identified a multitude of learning outcomes (summarized in Fig. 2). In general, learners reacted well to intraprofessional activities, their collaborative attitudes and mutual perceptions improved, and learners gained knowledge and skills necessary for intraprofessional practice. A small number of papers also reported positive changes in behaviour in clinical practice.

Many of the included studies described unidirectional learning, especially in situations when residents rotated in another specialty or received formal education from other specialists. This inequality in learning relationships has also been described in the field of interprofessional education by Baker et al. [21]. These authors cautioned that unequal power relationships threaten collaboration and learning and that learning in unequal positions only strengthens unproductive power dynamics. Similar findings were also reported in a recent ethnographic study on intraprofessional learning in hospital rotations for primary care residents [78]. This study observed that primary care residents often adapted the professional identity of the medical specialists and did not express their own professional identity during the rotation, thereby limiting opportunities for the specialist trainees to learn from their expertise in a reciprocal manner. Another possible explanation for the reported unidirectional nature of the learning is that the learning was in fact reciprocal but was not recognized or described as such due to a lack of awareness or a different research focus. Visiting learners often engage in intentionally organized and guided novel learning experiences, whereas the ‘experts’ interact while engaging in their everyday work. Billett explains that learning occurs through engagement in everyday workplace activities, but this learning is more implicit than in novel situations [77, 79, 80]. Furthermore, learning is dependent on how individuals elect to participate in work practices and what they construct from that participation, which is likely different between novices and experts [77, 79, 80]. The complexity of modern patient care results in growing interdependence between health professionals, and overcoming professional silos is necessary for all health professionals dealing with modern care challenges [1–4, 7]. It is, therefore, imperative that bidirectional intraprofessional learning of both residents and expert professionals is promoted in order to achieve high-quality, patient-centred care [7].

It could be argued that the reported positive results were due to the self-reported measures used in the majority of studies. As humans are poor at self-assessment, the actual change may well be less than what was reported [81]. Furthermore, many of the studies included in this review reported on activities with voluntary participation; in several studies the authors noted that this self-selected cohort may be more enthusiastic, which may have contributed to the positive outcomes found in these studies [43, 59, 75].

The self-reported nature of these findings does not imply meaningful changes did not occur. It has been well established that a person’s willingness to engage in and sustain self-directed learning efforts depends on their ability beliefs and motivation [82–84]. Additionally, one’s self-perceived accomplishments may also in itself act as a motivator for further learning [82]. The enhanced understanding of the importance of intraprofessional collaboration observed in the included studies suggests some extent of internalization of motivation, which has been strongly associated with behavioural change [83]. For these reasons, the self-reported outcomes described in the included studies do provide valuable insights into intraprofessional learning.

### Influencing factors

This review discussed a variety of influencing factors, which are to a large extent consistent with the influencing factors described in the literature on interprofessional learning, e.g. learner experience and enthusiasm, faculty attitudes, workload, stereotypes and negative perceptions, and health care and educational policies [12, 15, 16]. However, some of the influencing factors or aspects reported in this review have not been previously described and may be unique to intraprofessional collaboration and learning.

The findings in this review provide an insight into how high complexity of care can both facilitate and hinder intraprofessional learning. Previous studies emphasized that, whereas tensions in complex care can be highly productive in learning, the conflicts and stress that derive from these tensions may also cause professionals to retreat to their ‘safe’ professional silos in an attempt to preserve one’s self-esteem and dignity [4, 22, 85]. In line with these studies, our review reveals that complex intraprofessional care was considered to have the highest learning potential [52, 53], whilst one study reported that residents displayed avoiding behaviour after experiencing conflicts in care situations [45]. This review sheds new light on the influence of supervisors on this process. Billet highlighted how the pedagogic practices of experienced co-workers (e.g. supervisors) influence the quality of workplace learning experiences through direct guidance of learning and managing access to experiences [80]. Included studies reported that, in complex care situations, supervisors often restricted access for residents by diminishing them to observant roles or even completely bypassing them in intraprofessional communication [49, 52, 53]. Based on the findings of this review, we argue that ensuring adequate guidance of residents in dealing with complex care situations is of the utmost importance to fully harvest the learning potential of complex intraprofessional care.

Similar to the literature on interprofessional learning [12, 15, 16, 21, 86], we found that intraprofessional collaboration and learning were threatened by professional stereotyping and negative perceptions. The finding that supervisors displayed poor role modelling behaviour, were less reflective in collaboration and expressed more stereotypes than residents corroborates the idea that these intraprofessional biases develop through the socialization processes and professional identity formation in postgraduate training [2, 4, 20, 21, 86]. On a positive note, the results of this review suggest that participation in intraprofessional activities contributes to a positive learning climate and leads to improved attitudes. Consistent with the interprofessional learning literature [12], we found that these positive effects may fade if intraprofessional contacts are not maintained. Taken together, these findings implicate that repeated exposure to intraprofessional activities throughout postgraduate training is necessary to achieve a lasting impact on collaborative attitudes.

Included studies emphasized that awareness of learning opportunities and explicit reflection play a pivotal role in intraprofessional learning. The studies that observed behavioural change often involved individual or team reflection as part of the described activity or through the research methodology. These findings are in agreement with previous research which argued that guided team reflection is essential for collaborative practice and learning [87, 88]. The reported lack of faculty development in this area is unsettling, as previous research has established that improperly guided, superficial reflection may only consolidate pre-existing collaboration challenges and reinforce siloed professional identities [87].

Although there were many similar findings in studies describing intraprofessional activities within the hospital and across hospital boundaries, some of the barriers to intraprofessional learning seemed more prominent at the primary-secondary care interface, including stereotyping, physical distance, technological barriers and malalignment of competency frameworks [44]. It should be noted that these findings are likely context-dependent and therefore probably cannot be extrapolated to all settings, as the organization of primary and secondary care is highly heterogenous throughout the world. Nevertheless, we suggest that better alignment of primary and secondary care residency training curricula will likely enhance intraprofessional learning opportunities for residents during postgraduate training.

### Future research

The majority of studies in this review depended on self-reported perceptions of change and only a small number of studies reported on behavioural change or improvements in clinical practice. Several studies reported outcome measures from the perspective of one specialty, not considering the views and experiences of others involved. We suggest that future research should focus on obtaining more robust data through previously validated tools, more objective behavioural measures and reciprocal measurements. Reeves et al. [89] published guidance on how to improve the quality of studies investigating interprofessional education; we propose that these guidelines are equally suitable for studies investigating intraprofessional learning.

Second, this review found that the available literature does not provide an in-depth understanding of how intraprofessional learning takes place, and what works, for whom, and in which context. Further work is required to understand the mechanisms involved in intraprofessional learning and the interacting relationships between sociocultural environment, presage, process and product factors.

### Strengths and limitations

To our knowledge, this is the first review to examine intraprofessional learning in postgraduate medical education. This study provides a comprehensive overview of the learning outcomes and influencing factors reported in intraprofessional learning and provides relevant insights for future research and practice. Another strength of this study is the diversity of backgrounds and expertise in our research team, which included members with extensive experience both in clinical practice and research in postgraduate medical training, workplace learning, and inter and intraprofessional education, and members working in primary and secondary care, which allowed discussion and interpretation of the findings from different perspectives.

A limitation of this scoping review is that we did not perform targeted searches for grey literature for feasibility reasons. Furthermore, the terminology used for intraprofessional learning by authors in the medical education literature is highly heterogenous. Despites our efforts to cover the whole breadth of terminologies in our search strategy, it is possible that we omitted less frequently used terms. Due to these limitations, we might have missed relevant articles. However, given the breadth of methodologies, activities and specialties represented in this review, we believe that our results provide a good map of the learning outcomes and influencing factors of intraprofessional learning in postgraduate medical education. Per the scoping review approach, we did not explicitly aim to assess the quality of studies included, however important methodological limitations regarding self-reported outcomes and other research focus than intraprofessional learning were identified. Finally, we acknowledge that the low number of reported negative outcomes may reflect a publication bias.

## Conclusions

This scoping review provides an comprehensive overview of the evidence on intraprofessional workplace learning in postgraduate medical education. These findings support the high learning potential of intraprofessional activities. Moving forward, research should focus on (1) gaining a better understanding of the mechanisms involved in intraprofessional learning and (2) generating more robust evidence with more objective examination of changes in behaviour and performance in practice.

This review illuminates the multitude of factors that influence intraprofessional learning in the workplace, which can be used to develop targeted interventions to enhance intraprofessional learning. Building on the practical implications of this study, we present a series of recommendations for educational policy makers, program directors, residents, intraprofessional teams and any other person interested in strengthening intraprofessional learning in clinical practice (Table 3).

**Table 3:** Recommendations

**Individual level**	
• Encourage residents to set learning goals for intraprofessional practice to enhance awareness of learning.	
• Stimulate residents to seek exposure to intraprofessional collaboration and to faculty role models from different specialties to enrich learning by providing intraprofessional perspectives.	
• Pay explicit attention to bidirectional learning, especially in intraprofessional rotations. Encourage learners to contribute from their own professional roles in order to stimulate mutual understanding.	
• Ensure adequate guidance for residents in complex intraprofessional care, as complex care situations can be highly fruitful for learning, but may also result in conflicts.	
**Organizational level**	
• Facilitate resident participation in intraprofessional activities through the practical organization of work, and mitigate resident bypass, in order to ensure sufficient exposure to intraprofessional collaboration	
• Integrate time for individual and team reflection in clinical practice, in order to enhance awareness of and guide intraprofessional learning processes. Preferably, this reflection is facilitated by trained professionals.	
• Invest in faculty development, to better prepare faculty for their task as intraprofessional preceptor, facilitator, and guide, and to mitigate negative role models.	
• Promote fruitful learning climates and address unproductive hierarchy.	
**Strategic level**	
• Explicitly integrate and assess intraprofessional collaboration competencies in residency training curricula.	
• Ensure repeated exposure in residency training curricula to both formal and informal intraprofessional learning activities, as these seem to have a synergistic effect.	
• Align and coordinate residency training curricula of different specialties, especially for closely-related specialties.	
• Health care policy and funding structures should support intraprofessional learning for collaborative practice.	

## Supplementary Information

**Additional file 1.** Sample search strategy (PubMed).

## Data Availability

The datasets generated and analysed during the current study are available in the DANS EASY repository, 10.17026/dans-zb5-2hfg.
