# Supplementary material for: Intraprofessional workplace learning in postgraduate medical education: a scoping review
Source: BMC Med Educ. 2021 Sep 7;21:479. doi: 10.1186/s12909-021-02910-6 (PMC8424991; doi:10.1186/s12909-021-02910-6)
Supplement: Supplementary file 1 — Additional file 1. Sample search strategy (PubMed). [file 12909_2021_2910_MOESM1_ESM.docx]

**Additional File 1: Sample search strategy (PubMed)**

**Postgraduate medical education**

"Education, Medical, Graduate"[Mesh] OR Graduate Medical Education [tiab] OR Graduate medical student* [tiab] OR medical graduate student* [tiab] OR postgrad* [tiab] OR post-grad* [tiab] OR resident [tiab] OR residents [tiab] OR residency [tiab] OR residencies [tiab] OR intern [tiab] OR interns [tiab] OR internship* [tiab] OR Specialty train* [tiab] OR specialist train* [tiab] OR specialization train* [tiab] OR trainee* [tiab]

AND

**Intraprofessional**

"Interprofessional Relations"[Mesh:NoExp] OR "Interdisciplinary Communication"[Mesh] OR Intraprofession* [tiab] OR intradisciplin* [tiab] OR intra-profession* [tiab] OR intra-disciplin* [tiab] OR intersectoral [tiab] OR inter-sectoral [tiab] OR cross-disciplin* [tiab] OR cross-boundar* [tiab] OR boundary crossing [tiab] OR boundaries crossing [tiab] OR cross boundar* [tiab] OR inter-disciplin* [tiab] OR interdisciplin* [tiab] OR interprofession* [tiab] OR inter-profession* [tiab] OR transboundar* [tiab] OR trans-boundar* [tiab]

AND

**Learning and education**

“Learning”[Mesh:NoExp] OR Learning [tiab] OR learn [tiab] OR learner* [tiab] OR “Education”[Mesh:NoExp] OR “education” [Subheading] OR Education* [tiab] OR training [tiab] OR trainee* [tiab]

**Filters**

English language

Publication date from 2000/01/01
